# On Diseases of the Bladder

**Published:** 1838-07-01

**Authors:** 


### ON DISEASES OF THE BLADDER. By *William Coulson*, Surgeon, 12mo. pp. 153, London, 1838.

Mr. Coulson is already well known to the profession, by his works on Diseases of the Hip-joint, and on the Deformities of the Chest and Spine. An indefatigable and able surgeon, his works are marked by a free spirit of inquiry, and an accurate acquaintance with pathology.

His object, as he states in the Preface to the present volume, has been to establish clearly, the distinction between the inflammatory diseases which attack the several coats of the bladder. It has appeared to him important that these affections should not be confounded under the general character of inflammation of the bladder, as not only are they distinguished from one another by their symptoms and progress, but as each of them requires an essentially different treatment. He has also treated of Irritation, Paralysis, and some other affections of the bladder, connected, as they are, with inflammation of the organ.

The volume is made up of ten Chapters. These are successively devoted to the consideration of:—Irritability of the bladder—Paralysis of the Bladder—Acute Inflammation of the Mucous Membrane—Sub-acute Inflammation of the Mucous Membrane—Acute Inflammation of the Muscular Coat—Chronic Inflammation of the Muscular Coat—Inflammation of the Peritoneal Coat, and of the surrounding Cellular Tissue—Fungus Hæmatodes and Cancer of the Bladder—Foreign Bodies in the Bladder.—Operation for Stone—Wounds and Injuries of the Bladder.

### 1. IRRITABILITY OF THE BLADDER.

Mr. Coulson very properly limits the application of this term to a frequent and often irresistible, desire to void the urine, sometimes, but not always, attended with pain, and not arising from inflammation, or any of the organic affections, of the bladder or prostate gland.

We need not particularize the symptoms of the disorder. The aggregate quantity of water passed does not much exceed that of a person in health—that quantity being calculated by Rye to amount, on an average, to forty, and by Dr. Prout to be thirty-two ounces in the course of the twenty-four hours.

In hysterical patients, the quantity is often considerable, and possesses the peculiar character designated by Dr. Bostock 'Aqueous', containing less than the usual proportion of solid matter, without any other change in the nature or relative quantity of its constituents.

Mr. Coulson has, in one instance, examined the body of a patient who laboured under this affection, and who was carried off by disease of the lungs. There was no appreciable alteration in the appearance or structure of the bladder or of any of the urinary organs.

" After long continued irritation, the bladder becomes diminished in size, and instead of containing a pint or upwards, it is incapable of holding more than two or three ounces at a time. Notwithstanding this contracted state of the bladder, if there be no stricture or disease of the prostate, its parietes are often thinner than natural." 4.

The causes of irritability of the bladder are numerous. Sometimes they are of an obvious character—such as pressure of the womb in pregnancy, stricture of the urethra, foreign bodies in the bladder. After the operation for stone, the bladder often remains in an irritable state.

Derangements of the digestive organs, by altering the constitution of the urine, not unfrequently give rise to irritability of the bladder. Thus, adults and children, more particularly, are, from eating fruit, very liable to this affection in the Summer. The urine, in these cases, contains an excess either of lithic acid, or of lithate of ammonia. For the same reason, gouty and rheumatic persons are subject to it. Mr. Coulson relates a case of this kind—a case by no means rare.

*Case.* "A gentleman, forty-two years of age, and subject to rheumatism, applied to me on the 11th of February, 1837, on account of a very frequent desire to pass urine, from which he had suffered for several years. There was a scaly eruption on several parts of the body, particularly about the elbows and knees, and he often felt severe pains in the hips and loins. The urine was very acid and scanty. I ordered him the following mixture:—℞. Infus. Diosmæ ℥xv.; Tinct. Hyoscyam. ʒiij; Potass. Bicarb. ʒiss; Extract. fluid. Sarsap. ʒiv; Cap. coch. ij. vel iij. ampl. ter in die. And I gave the following pill at bed time: ℞. Pil. Hydrarg. gr. iij; Pulv. Rhœi. gr. ij. On the 3rd of March, the irritability of the bladder was much lessened, and the eruption improved. I then gave the decoction of the Pareira Brava in the day, with a grain of the acetous extract of colchicum at bed time. His complaint was much relieved, but not cured." 7.

Last year we attended a troublesome case of the same description. The patient was a young Oxonian, of indolent habits, and rather sedentary in his pursuits. The irritability of the bladder was considerable, and he suffered from so much pain in the urethra that we twice sounded him, with the suspicion that the bladder contained a stone. The urine was very acid, on two or three occasions small particles of red sand had been passed, there was a disposition to acné, the habit was leuco-phlegmatic, and both parents had suffered from gout or rheumatism. This gentleman had been for some months under the care of the medical attendant of the family in the country, and had derived some, but not material benefit from his remedies. We prescribed cupping on the perineum—the occasional use of the warm bath—regular exercise and regimen—regulation of the secretions by small doses of the blue pill with ipecacuanha, and alkaline aperients with colchicum. We tried the uva ursi and the buchu, but the liquor potassæ with tincture of henbane, and the blue pill with alkaline aperients, combined with occasional recurrence to cupping on the perineum, were successful, after two or three months, in subduing all the symptoms.

"Sometimes it is produced by taking for too long a time alkaline remedies; and, in this case, the urine is of course alkaline. I was consulted for irritability of the bladder by a gentleman, whose urine was alkaline, but whose appearance and state of constitution did not at all lead me to expect this condition of the urine. On inquiry, I found that he had for a long time been taking the sesquicarbonate of soda in large doses. I ordered him to discontinue the use of this medicine, and he soon recovered. The altered state of the urine is the immediate cause of this complaint; and, in these cases, the condition of the urine should especially engage our attention." 5.

The fact is, that alkaline as well as very acid urine, stimulates the bladder, and gives rise to irritability of the organ. Some persons have, without any obvious state of disease, a condition of urine either neutral or slightly alkaline. These persons are never strong, and in too many instances, organic disease of the kidneys succeeds. Slight causes produce in such persons an alkaline state of the urine, and whenever this obtains they have irritation of the bladder.

Independently of an acid or an alkaline condition of the urine, irritation in the bowels will induce irritation in the bladder. Hæmorrhoids, scybala in the colon or rectum, even irritants in the stomach and the small intestine, will give rise to this sympathetic state of the bladder.

Mental emotions occasion the disorder. More physical affections of the nervous system do so too. Sir B. C. Brodie alludes to this fact. "An elderly man, for example," says he, "complains of frequent attacks of giddiness. In walking, his head turns round, so that he is in danger of falling; and this symptom probably arises from an altered structure of the arteries of the brain, causing an imperfect state of cerebral circulation. Not unfrequently this is attended with an irritable state of the bladder; and although the urine is of a healthy quality, and the bladder itself is free from disease, the patient is tormented by a constant micturition, voiding his urine without pain, but at short intervals, and in small quantities at a time."

"I have known," says Mr. Coulson, "irritation supervene on paralysis of the bladder. A gentleman, during an attack of typhus fever, nine years ago, was seized with an inability to pass urine, requiring it to be drawn off twice a day. However, as he gained strength, the power of voiding the urine returned, but the desire to pass it became then so frequent as to compel him to go every half hour. He consulted me for this symptom on the 25th of November, 1835. There was no disease in the urinary organs and his general health was good, with the exception of occasional rheumatic attacks. The urine was very acid and scanty. I ordered him a grain of the acetous extract of colchicum at night, and ten grains of bicarbonate of potass, seven of sesquicarbonate of soda, and four of the nitrate of potass, twice or three times a day, soon after his meals; and by these means the urine became more abundant and less acid; but the frequency of passing the urine continued the same. I then tried the infusion of diosma, the decoction of the Pareira Brava, and various preparations of steel, for the relief of this symptom, without success." 9.

Disease of the kidney, even irritation of it, not unfrequently produces excessive irritation of the bladder. The symptoms of the affection of the kidney may be masked, or rather irritability of the bladder may be its only symptom. Unless the urine is altered in its qualities, there are no means of determining the renal origin of vesical irritation. In some instances, the urine is so altered, and albumen may be found in it by the usual re-agents. Morgagni has recorded a case of this kind, and Dr. Prout and Sir B. Brodie have strongly drawn attention to it. Mr. Henry James Johnson has published some cases of the same description, and altogether, the body of evidence on this point is both considerable and conclusive.

In some instances, it is difficult to say on what cause the complaint depends. Mr. Coulson relates a case of this description, and there are few surgeons who have not seen similar ones.

*Case.* "A gentleman, sixty-five years of age, of a good constitution and regular habits, consulted me on the 4th of December, 1835, on account of a very frequent desire to pass his urine, unaccompanied with stricture or disease of the prostate, or any other affection that I could detect. Thirty years ago he applied to Mr. Jesse Foot, for the same complaint; and the lotura vesicæ was then tried on him without any relief. This symptom has continued ever since, being aggravated in cold weather, and on any excess in living or derangement in the health. In other respects, he is quite well." 11.

*Treatment.*—Irritability of the bladder being a complaint produced by various causes, it is indispensable to endeavour to detect the cause in every individual case, and, having detected, to remove it. The treatment of the disorder must consequently vary, and no single plan can apply to all forms of it.

*a.* In gouty and rheumatic subjects, where the urine is generally acid and scanty, and red sand is often passed, the alkalies should be administered; and a good form for their exhibition is a combination of the potass, soda, and nitre. Mr. Coulson subjoins a formula for this:

"℞. Bicarb. Potass. ℥j.; Sesquicarb. Sodæ, ʒvj.; Potass. Nitrat. ʒij. As much of this powder as can be put on a sixpence, to be taken twice or three times a day, in water, soon after a meal."

Dr. Prout, as is well known, recommends the carbonate of potass, in preference to the carbonate of soda, because the soda, under certain circumstances, enters into combination with the lithic acid, forming an insoluble salt, as bad as the lithic acid itself, whereas the lithate of potass is perfectly soluble, and if this combination should take place, it will pass off dissolved, in the urine. We have found the combination of the liquor potassæ with nitrate of potass, and infusion of rhubarb answer well. Mr. Coulson proceeds to remark that the acetous extract of colchicum is an useful addition to the alkalies. The dose may be from one to two grains in the form of pill, at bed time. Not a bad mode, we think, of exhibiting colchicum is in combination with the sulphate and carbonate of magnesia, or, in the common seidlitz draught. If, as often happens, the digestive powers are weak, some light bitter infusion may be given before meals.

"In these, and indeed in all cases, the strictest attention should be paid to diet; vegetables and fruit should be avoided, as well as wine, spirits, and all fermented liquors. In some constitutions, notwithstanding the acid state of the urine and the deposition of a large quantity of the lithate of ammonia, the alkalies disagree, producing restlessness, giddiness, and uneasiness about the region of the stomach.

I recently saw, with Dr. Prout, a gentleman who had great irritability of the bladder and whose urine was very acid and deposited great quantities of the lithate of ammonia, but who could not bear even small doses of alkaline remedies.*" 15.

*b.* Mr. Coulson goes on to remark that, if the patient is of a nervous temperament, and the urine alkaline, a different plan must be adopted.

"The dilute mineral acids,† combined with the decoction of Pareira Brava,

---

* "In such cases the following mixture may be given. ℞. Spirit. Ammon. Aromat. ʒij.; Spirit Æther. Nit. ʒij.; Tinct. Hyoscyam. ʒij.; Mist. Camph. ℥v. A fourth part to be taken three times a day."

† "℞. Acid. Nitric. dil. ʒj.; Acid Muriat. dil. ʒss.; Aquæ distillat. ℥viij. Two table spoonsful to be taken three times a day."

should be administered, and every thing that has a tendency to lower the system, as attention to business, study, or anxiety, should be studiously avoided. In other cases, where the urine is neutral, the Extract of the Uva Ursi,* combined with extract of Hop or Hyoscyamus, may be taken, with opiate suppositories,† or injections with some drops of the Liquor Opii Sedat. according to the severity of the symptoms, may be administered. The decoction of Uva Ursi,‡ and the infusion of wild carrot seeds,|| will occasionally give great relief. But, in my experience, no medicine has been so often successful in irritability of the bladder, as the Diosma in the form of an infusion.§ I could cite several cases, where it has succeeded after other medicines had failed. A young gentleman, æt. 21, applied to me, May 25th, 1834, on account of an affection of the bladder; and said that, for the last eleven or twelve years, he had experienced great difficulty in retaining his water for any length of time, being obliged to leave any company in which he was, once or oftener in the hour. The moment the desire to pass the urine came on, the water passed away involuntarily, unless the desire was immediately complied with. This irritability of the bladder was always very much aggravated after taking malt liquors, wine, spirits, and on exposure to cold, and had considerably increased during the last twelve months. He was very susceptible of cold, complained of pains down the inside of the thighs, but he had no pain in the region of the loins on pressure nor over the bladder, and his general health had not suffered. The urine was light colored, and neutral in its character. After trying the various preparations of steel, the decoction of the Pareira Brava, henbane in different forms without success, I ordered the infusion of Diosma, which he took for some time with great benefit." 17.

We may add our testimony to the value of the buchu in many cases of irritable bladder. We have found it of service in cases where the urine was acid, as well as in those where it was alkaline. We have also found it useful in cases which we have suspected to be incipient disease of the kidney. The patient has extreme irritability of bladder, the urine is acid, straw-coloured, and rendered slightly opaque by nitric acid—no disease can be detected in the bladder—there may or may not be some pain in the region of the kidney—and the general aspect is that of impending, if not actual organic disease. In such cases we have seen very marked advantage from regulation of the secretions, the infusion of buchu with henbane and perhaps alkalies, and local depletion or counter-irritation.

We cannot say that we have found as much benefit from the uva ursi, as other surgeons have derived from it. We have prescribed it, and we have

---

* "℞. Extract. Urvæ Ursi. gr. v. Extract. Humuli vel Hyoscyam. gr. iij. Two pills to be taken three times a day.

† "℞. Pil. Saponis cum Opio, gr. vij. To be introduced within the rectum at bed time."

‡ "℞. Folior. Uvæ Ursi ℥j.; Aquæ ferventis ℥xx.; coque ad ℥xvj. A third part of a pint to be taken three times a day."

|| "℞. Seminum Dauci Contusorum ℥j.; Aquæ ferventis ℥xvij.; Macera per horas iv.; dein cola. A third of a pint to be taken three times a day.

§ "This is to be prepared according to the formula in the London Pharmacopœia. If there be scaly eruptions, and the urine very acid, the following form will be found of service: ℞. Infus. Diosmæ ℥vij.; Potass. Bicarbon. ʒj.; Tinct. Hyoscyam. ʒiss.; Extract. Fluid. Sarsap. ʒiv. Two table spoonful to be taken three times a day. If the urine be not very acid, the alkali must be omitted."

concurred with the prescription of it in consultation, but we have usually been disappointed in its operation.

Mr. Coulson does not allude to cupping on the perineum, but we have seen advantage from it. We have seen, too, the warm bath serviceable, as well as the external application of opium and of belladonna.

On the whole, we should say, from what we have seen, that cautious local depletion—sedatives administered by the mouth, by the rectum, and by the skin—the buchu—alkalies in most instances—and regulation of the secretions—are the remedies which we have seen of greatest service.

*c.* Mr. Jesse Foot recommended the injection of warm water into the bladder in cases of irritability of this organ. We have tried it in three or four instances, but cannot say that we found it of advantage. We have tried the injection of aqueous solutions of opium, of aqueous solution of conium, and, in one instance, of a weak solution of the extract of belladonna. We thought that all were of a little service at first, but, after a time or two, the operation was rather productive of irritation than otherwise.

*d.* In the irritability of the bladder, says Mr. Coulson, which is met with in young females, just at the time when menstruation may be expected, or when some irregularity in this function has occurred, the preparations of iron are of great service. The ammoniated or the muriated tincture of iron, given in some light bitter infusion, willl be found serviceable ; and if the bowels be costive during the use of this remedy, the compound decoction of aloes should be daily administered. The bowels should be kept well open, for the symptoms of the disease are invariably aggravated when the bowels become costive. If there be much hysteria, the tincture of valerian, combined with the vinum aloës, may be also tried with benefit.

We have seen the shower bath very useful in checking the irritability of bladder which occurs in nervous girls, in combination with "hysterical" symptoms. The surgeon should always discourage the idea of attributing importance to the malady. To pay much attention to it, is often to aggravate it.

"Incontinence of urine in children usually depends on an excitable state of the bladder, or an altered condition of the urine. It occurs during sleep, and the urine is often passed off voluntarily, under the influence of a dream. In other cases it is more of a passive character, and passes involuntarily. In some cases, this involuntary passing of the urine continues by day as well as by night, and then the patient suffers more or less from this complaint during the remainder of his life. In such cases the children should be waked up in the night, twice or oftener, for the purpose of passing the urine; and they should not be allowed to take late meals or much liquid for some time prior to retiring to rest. In fact, they should not, at any time, be allowed to take much liquid. Lying on the back in bed tends to keep up this complaint, and should be guarded against. Cold bathing, preparations of iron, and other tonics, will be found of great use. If the complaint continues after puberty, the tincture of cantharides is often very serviceable.

It is frequently connected with a weak and scrofulous state of constitution; and in these cases, all remedies are often unavailing. I lately saw a child six or seven years old, with a large head, pale countenance, bad teeth, prominent sternum, large abdomen, and emaciated extremities, who had suffered from incontinence of urine from its earliest infancy. All kinds of remedies had been tried without any success. I may just observe that, in this case, as well as many others, there was great irritability of the bladder during the day-time, and, unless the desire to pass the urine was attended to, it flowed off involuntarily. The urine was very acid.

Incontinence of urine is sometimes the effect of stricture in the urethra, and will subside on the cure of the stricture. In other persons, particularly stout females, the urine will flow involuntary on lifting a weight, or coughing, or any violent exercise. In these cases there is no pain, no blood in the urine, no desire to make water often, simply the involuntary flow on exertion, the action of the diaphragm, and abdominal muscles overcoming that of the sphincter of the bladder. In females, it often occurs after distention of the urethra for the extraction of calculi or foreign bodies, and occasionally after difficult labours." 21.

Incontinence of urine in children usually disappears, so far as we have seen, with growth, independently of the exhibition of medicines. But in troublesome cases, we have seen the greatest benefit from the tincture of cantharides. We had lately under our care a lad of 17 years of age, who had laboured under incontinence of urine since infancy. Many remedies had been tried, but none had been of use to him. We prescribed the tincture of cantharides, beginning with a small dose, and gradually increasing it to a drachm thrice daily. This cured the patient in the course of four months.

On the whole, we would say that the study of the causes, and the determination of the treatment of irritable bladder, are of great practical consequence. We recommend the subject to every surgeon who is anxious to be successful in his management of a troublesome disorder.

### II. PARALYSIS OF THE BLADDER.

We need not dwell on the symptoms. We may remark, however, what has indeed been frequently remarked before, that when the bladder becomes exceedingly distended, the urine usually dribbles away, and the complaint may be mistaken for incontinence. Mr. Lawrence relates a case so much in point that we extract it.

*Case.* " It happened to me, a good while ago, to be sent for, to see a gentleman labouring under an affection of the bladder; and the medical attendant who had lately seen him, mentioned that the case was one of great irritability of the bladder—that it would hold no water at all—the urine passing off as fast as it came into it. He said he had been doing all he could to get the natural power of retention of the bladder restored; he had directed the patient to take diluent fluids; in short, he had done all he could to prevent it; but still the water ran off. It appeared to be a singular case. I put my hand under the clothes upon the abdomen; and I felt the fundus of the bladder forced up a good way above the umbilicus: I said I had brought a catheter with me, and that I might as well introduce it, to see if there was anything in the bladder. I introduced it; and about five pints of urine immediately flowed off. The fact was, the bladder had been allowed to distend in this way for about five days before I saw him; and the consequence was, that that gentlemen never recovered the natural power of emptying the bladder afterwards; but he, after a certain time, acquired the art of introducing the catheter, which he still employs. He can introduce it, and let off the water whenever he finds a desire to do so; but he never has been able to empty the bladder by the natural powers since that time." 30.

The causes of paralysis of the bladder are examined in detail by Mr. Coulson.

*a.* Injuries or diseases of the spine are a common cause of it. In some cases, the urine becomes highly acid. In the majority of instances, it grows extremely alkaline, is voided of an ammoniacal odour, and deposits, when cooled, a large quantity of adhesive mucus. After some time phosphate of lime is detected in it, and the mucus is tinged with blood, which subsequently appears in larger quantity and coagulated. It admits of question whether the urine is secreted alkaline by the kidney, or becomes alkaline in the bladder. The matter is undecided, though it is of consequence to determine it. If the change occurs in the bladder, the regular evacuation of the latter by the catheter may prevent the alkalescence and its consequences.

*b.* Diseases of the brain operate, we may suppose, in a similar manner with injuries and diseases of the spine in producing a paralytic state of the bladder. The complaint, as Mr. Coulson observes, often attacks elderly persons, particularly gouty and rheumatic subjects, and is the result of general diminution of muscular and nervous power, the bladder being incapable of obeying the will with the same facility as before, and being less sensible to the stimulus of the urine.

*c.* Neglecting to expel the urine when it is accumulated is another cause of paralysis. A person, says Mr. Coulson, not being conveniently situated for emptying his bladder, neglects the first call, and allows it to become distended; the desire perhaps goes off; a large quantity of water accumulates; the bladder rises up to the umbilicus, or even higher; and when he attempts to empty it, he finds he is totally unable to do so, and that he cannot void any water at all.

Patients ought never to resist the first desire to make water: for, in not obeying this inclination, the bladder distends; the elongated fibres lose more and more their sensibility; the desire to make water passes off; the retention, which, in the commencement, was only partial, then becomes complete; and there is no stimulus sufficiently strong to excite the bladder to expel the urine which it contains.

*d.* Paralysis of the bladder not unfrequently occurs in persons under thirty-five or forty years of age, whose constitutions have been seriously impaired. The patient, perhaps, complains of pain in the head, or some part of the back, weakness in the loins, and inability to walk firmly, flatulence, or a sense of fulness in the region of the epigastrium, and he looks and feels as if threatened with some impending mischief. If these symptoms be not relieved, paralysis of the lower extremities follows, and his bladder partakes of the affection. Mr. Coulson states that he has known paralysis of the bladder occur where the state of health was good.

"With Mr. Dunn, of Norfolk Street, I, last year, attended a gentleman, about thirty years of age, extremely nervous, who had been labouring under considerable mental excitement. We were sent for to this gentleman, suddenly, on account of retention of urine: the urine was drawn off by the catheter, and this was repeated twice a day for ten days, at the end of which time the power of the bladder returned, and he was able to make water himself. The retention arose entirely from a paralysed state of the bladder, owing to diminution of nervous power: there was no stricture, or gonorrhœa, or local impediment, to the passage of the urine." 33.

*e.* Paralysis of the bladder may result from severe injuries of the lower extremities. It is not difficult to explain the fact of its occasionally following the operation for hæmorrhoids.

*f.* It now and then happens from the use of opiate suppositories or injections.

*g.* Pressure on the urethra or neck of the bladder may give rise to it. It may consequently be occasioned by distention of the rectum, however induced, by abscess near the neck of the bladder, by various tumors in the pelvis. Mr. Coulson dwells on the effects of retroversio and prolapsus uteri upon the bladder.

" There are two periods in pregnancy when women are particularly exposed to retention of urine—about the fourth month of pregnancy, and at the time of confinement. To have an exact idea of this state, it must be recollected, that, in the first month, as before conception, the womb continues concealed in the pelvis; that it does not mount above the cavity till the fifth month, and sometimes even later; that until that time, its size and weight having progressively increased, it descends lower towards the vagina, and in the manner of a wedge, presses posteriorly the rectum, and anteriorly the neck of the bladder, and the urethra against the symphisis of the pubes, even to such a degree, as to stop the opening of this canal.

The displacement of the viscera, which so often gives rise to retention of urine are retroversion of the womb and prolapsus of this organ, of the vagina and of the rectum. If we examine the intimate connexion of the bladder both with the womb and vagina in the female, and with the rectum in the male, it is clear that the parts cannot be displaced without drawing with them the bladder; and that, in this displacement, whatever may be the contractile force, it cannot entirely expel the urine which it contains.

In the prolapsus and retroversion of the womb and vagina, and of the rectum, the posterior part of the bladder, instead of being carried upwards and forwards, is drawn downward and backward, and the curve of the urethra is entirely changed. Instead of presenting a concavity beneath the pubes, as in retroversion, the bladder presents there a convexity, a derangement which must not be lost sight of in the introduction of the sound." 36.

*Morbid Appearances.* These are easily summed up. They are dilatation of the bladder, attenuation of its coats, and a pale white appearance of the mucous membrane. When the urine has grown alkaline, and chronic inflammation of the mucous membrane of the bladder has been set on foot, then we have its characteristic appearances—appearances on which we need not dwell at present.

*Treatment.* We shall quote what Mr. Coulson says upon this subject.

" The first and immediate step required, is to draw off the accumulated urine by means of a catheter; and the operation should be repeated twice, or even oftener, in the twenty-four hours (at intervals sufficiently short to prevent over distension,) until the bladder has recovered its contractile power. If the complaint has not arisen from organic mischief, and the patient is not very old, recovery may be expected to take place; and, as the bladder recovers its tone, the patient says he fancies he could pass a little urine; and, on making the effort, voids some, either drop by drop, or in a small stream. As the recovery begins to take place, the catheter should be less frequently employed, until its use be entirely superseded. In other cases, particularly in elderly persons, the bladder never recovers its tone, and the use of the catheter will be required as long as the patient lives. In the paralysis of the bladder dependant on disease or injury of the spine, the catheter should be passed with the greatest care and caution, so as, on the one hand, to avoid injury to the mucous membrane, and, on the other, completely to empty the bladder.

These are points of the greatest importance, which, perhaps, have not always been sufficiently attended to in practice. Dr. Burne mentions, in the paper, already quoted, a case in which so long as the bladder was completely evacuated, the urine retained its natural character, and the bladder remained free from disease.

To prevent any injury to the mucous membrane, a gum elastic catheter had better be used; and, at the time of passing it, pressure on the pubes should not be used, nor should I advise the catheter to be kept in the bladder, but it should be introduced on each occasion when the urine requires to be drawn off. Cases are on record where abscesses in the bladder and ulcerative perforations have been the result of the point of the catheter coming in contact with the bladder.

I need scarcely observe, that if, from any circumstance, as impermeable obstruction of the urethra or enlargement of the prostate, the catheter can not be introduced, the life of the patient must not be endangered by delay: but the bladder must be punctured." 40.

We would observe, that the regular introduction of the catheter is certainly not sufficient to prevent the alkaline condition of the urine, in cases of injury of the spine. We have in several instances seen this condition of the urine established, when the catheter was introduced with regularity.

In a case of compound fracture of the leg, which was under our care a few years ago, the patient was suddenly attacked with paralysis of the bladder, and the urine rapidly grew alkaline and loaded with adhesive mucus. A few injections of water with a little nitric acid removed these symptoms as rapidly as they had come.

The constitutional treatment must vary with the cause of the disorder.

Mr. Coulson alludes to the paralysis of the bladder so apt to occur in hysterical girls. He wisely recommends their being let alone. When they are, the retention of urine seldom lasts, but if the catheter and surgeon are always at hand, it may go on till the latter is tired.

### III. Acute Inflammation of the Mucous Membrane of the Bladder.

Mr. Coulson presents a concise account of the symptoms of this affection. But they must be tolerably familiar to our readers. If the inflammation is not arrested, ulceration of the mucous membrane occurs, and may proceed to the destruction of the whole mucous membrane. The symptoms of this alteration are not decisive, unless there are fæces passed by the urethra. But, as Mr. Coulson remarks, there is always reason to suspect it when disease of that organ has been of long continuance, when the pain is increasing and extensive, and when pus is distinctly detected in the urine. The ulcerative process is attended with constant pain and irritation, keeping up the desire to void the urine, which is never suffered to accumulate; at the same time, increased difficulty and increased pain generally attend the passing of it.

Ulceration may cause perforation of the parietes of the bladder and extravasation of urine. But, usually, the previous effusion of lymph has glued the neighbouring parts together, and escape of the urine is prevented, or a communication is established between the bladder and the ileum, or sigmoid flexure of the colon, or the rectum. In the former case, the fæces pass into the bladder and through the urethra—in the latter case, the urine is voided by the rectum. Mr. Coulson relates an instance in which there was stricture at the sigmoid flexure of the colon, and a communication existed between the bowel above the stricture and the interior of the bladder. The patient passed fæces with the urine during life. We have related in a former number of this Journal a case in which the cæcum communicated with the bladder by means of a circuitous sinus opening into each, and passing from one to the other. In this case no fæces were discharged by the urethra.

The more usual course of the disease, is, that ulceration gradually extends to the whole of the membrane, which is destroyed, and then the muscular structure is shewn better than any dissection can represent it. In the progress of the ulceration, disease commonly manifests itself in one of the kidneys, and, as far as Mr. Coulson's observation has gone, in the left kidney. This is indicated by rigors, sickness, more or less pain in the loins, albuminous, and purulent urine, tinged, perhaps, with blood.

"Attempts have been made to determine, by the qualities of the pus, whether it has been secreted from the bladder, or has passed from the kidney.

'When nearly pure,' says Dr. Prout, 'aud unaccompanied by mucus, or when it contains blood, it may be supposed in general to be derived from an abscess. Most frequently, however, it is accompanied by mucus: indeed, mucus and pus are so nearly related as to run into each other imperceptibly; and when the mucus is in excess and has preceded the pus, we may almost always conclude that some portion of the mucous membrane lining the urinary organs, is the common source of both," I may observe, that when pus and mucus exist in small quantities in the urine, it is difficult to distinguish one from the other. Pus, however, when well marked, may be distinguished from mucus by being composed of particles. Hence, when diffused through a fluid, the latter is rendered opaque, though, upon standing, the pus subsides to the bottom of the vessel, in a state more or less pulverulent, and the fluid assumes its transparent character, but it also mixes more readily with the urine. If in urine pus be present with mucus, it is found lying on the latter, and presents a much yellower tint; it is also quite opaque; whereas mucus is more or less transparent.' 52.

We need not speak of the appearance of inflamed or ulcerated bladder, when examined after death. We may merely observe that the inner membrane is sometimes covered with coagulable lymph, which has been found projecting into the cavity of the bladder, and, during life, portions of it have been occasionally separated.

One of the kidneys is usually in a state of ulceration, and contains pus, the ureter being inflamed in its whole course, and ulcerated at its vesical extremity.

This affection, says Mr. Coulson, may be confounded with inflammation of the muscular structure, but in the latter case there is not the power of passing the urine, and the desire to void it is less frequent, not being experienced until a good deal of urine is accumulated in the bladder, and then coming on in violent paroxysms. Neither is there the burning sensation along the urethra which is felt when the mucous membrane is affected.

This disease is very likely also to be mistaken for stone. The uneasiness in the bladder, the frequent desire to make water, and the passage of blood with the urine, are symptoms of stone as well as of this complaint. But in stone the pain is principally experienced after the bladder has been emptied, whereas, in acute inflammation of the mucous membrane of the bladder, the pain is most intense when the bladder contains urine, and subsides when it is empty: in stone, larger quantities of blood are passed than in this disease, and the urethra is seldom so irritable.

*Treatment.* Confining his attention to inflammation of the bladder, uncomplicated with any other organic affection of consequence, Mr. Coulson lays down the following plan of treatment:

"Blood should be taken at the commencement by the application of leeches to the hypogastric region, and they should be repeated so long as the severity of the pain continues, and the strength of the patient will allow. Commonly, however, the loss of much blood cannot be borne. The most valuable remedy at the early stage is morphium, or opium, (I prefer the former), given in sufficient doses to allay the pain about the bladder and along the urethra, as well as the frequent desire to pass the urine. These are the most distressing of the whole class of symptoms; and if unmitigated, they soon wear out and exhaust the strength of the patient; but if only a few hours' intermission be obtained in the day, some chance may exist for the recovery of the patient.

In addition to the internal use of opium, or morphium, anodyne injections, or suppositories, should be exhibited at bed time, and great relief will be experienced from their use. Some practitioners recommend the injection of oil and opium, and other remedies, into the bladder, by means of a gum elastic catheter; and in one of my patients, this plan had been tried at the suggestion of an eminent physician, prior to the patient being placed under my care, but no benefit was derived from this treatment. In fact, the pain and irritation which are experienced from the introduction of any instrument along the urethra, are so severe, as to deter me from employing this plan; and unless there be retention of urine, which is very rare in this form of disease, the use of the catheter, sounds, and bougies, should be particularly avoided.

It will be advantageous to employ counter irritation above the pubes; and the hip bath at night will be found very serviceable.

In the treatment of these cases, however, we find, that no remedy, opium or morphium perhaps excepted, long retains any influence over this complaint. The practitioner must be armed with a variety of agents, so as to be able to substitute one for another when it loses its effect. An infusion of diosma in the proportion of an ounce to a pint of water, the decoction of wild carrot seeds and parsly breakstone, small doses of copaiba and essential oil of cubebs, infusion of hops and the alkalies, will, all in their turn, be found useful." 58.

Mercury, observes Mr. C., is not of use in this form of inflammation, except at its commencement. Certainly we have found benefit from the combination of small doses of calomel, James's powder and opium, when the inflammation was acute.

The diet of the patient should, of course, be of the lightest and blandest description. He should not take diluents to the extent of increasing, to any material amount, the urinary secretion. The patient should be kept quiet, in rather a warm temperature, and he should remain, we would add, as much as possible in the horizontal posture.

Mr. Coulson has not alluded to a remedy which we have seen of much service, cupping on the perineum, or the application of cupping-glasses over leech-bites in that situation. We have found much advantage from directing the frequent repetition of the operation, taking care to abstract very small quantities of blood (from an ounce to three or four) and conjoining several dry cups with the wet ones. The acute inflammation of the bladder which occasionally follows gonorrhœa is much benefited in this manner.

*Prognosis.* This is very unfavourable, if the ulcerative stage has once set in. If females are affected (and they are more prone than males to this disorder), pregnancy relieves the symptoms, which return after delivery.

Mr. Coulson relates several cases of inflammation and ulceration of the bladder. They illustrate the preceding observations and descriptions.

### IV. Subacute Inflammation of the Mucous Membrane of the Bladder.

Men, says Mr. Coulson, are more subject to this complaint than females, and elderly persons than young ones. In some countries, the disease is so uncommon, that Hoffman calls it *morbus rarissimus.* In others, it appears more frequently, and something like it has been known to assume an epidemic character.

From the characteristic discharge of mucus in this disorder, it has received the name of *Vesical Catarrh.* This is accompanied by a sensation of heat in the bladder, extending along the urethra, weight in the perineum, shooting pains towards the anus, and a frequent desire to void the urine, although not to so great a degree as in the acute form.

Sometimes the symptoms are very mild, and cause but little inconvenience ; at other times, the disease assumes a serious character and even proves fatal, particularly in old and weak persons. Then, the heat in the bladder and urethra are converted to a scalding ; and the desire to make water, becomes more frequent, is attended with violent straining efforts to void it, and retention often takes place, being usually caused by clots of mucus blocking up the passage. On the urine being drawn off, the symptoms are relieved for a time, but return on the filling of the bladder.

The patient is very restless and uneasy ; there is great thirst ; the bowels are irregular, generally very costive or relaxed ; there is pain round the anus and in the region of the loins ; great prostration of strength and wasting of flesh ; and the patient at last dies completely exhausted.

The quantity of mucus secreted varies. In some recorded cases several pounds have been passed in the twenty-four hours.

The mucus is sometimes like panada, and, on being shaken, colours the urine without flakes. Small quantities, however, usually render the urine muddy, pale, and flaky, and gradually settle to the bottom of the vessel. It is commonly very glutinous, and may run like a rope from one vessel into another. Occasionally it adheres to the bottom of the pot, and cannot be poured out of it. Sometimes it is transparent, white, yellow, green, with streaks of blue, often without smell ; sometimes on the contrary, dreadfully fætid.

" When the properties of the mucus are but little changed, it diffuses itself throughout the urine for a time, and renders the urine turbid, and of a whitish colour, but afterwards subsides to the bottom, and leaves the urine to assume its usual colour. But it is commonly thick, viscid and ropy, and sinks to the bottom of the vessel at once ; in this case the urine is of a dark brown colour, and is either neutral or alkalescent. The urine, however, is usually acid at the commencement, and continues so until the quantity of mucus secreted is great: in this case especially, if the patients are very feeble, the urine is alkaline or neutral." 75.

The mucus may even obstruct the urinary passage, and so occasion retention.

On examining the bladder after death, the mucous membrane, in slight cases, is inflamed and presents here and there red spots, as if blood had been effused beneath the surface, while some are seen of a darker colour, almost amounting to black. Sometimes it is abraded, particularly around the darkest patches; and, in some rare cases, it has been entirely removed so as to leave the muscular fibres exposed. In severe cases the muscular fibres of the bladder are hypertrophied, and occasionally covered with calcareous deposits. In some cases of stone, an abscess is formed in the thickened coats of the bladder, and the mucous membrane is found in a gangrenous state. The kidneys are more or less affected, either simply inflamed or ulcerated.

The exciting causes of catarrh of the bladder, are thus enumerated by Mr. Coulson:—stone, enlargement of the prostate, exposure to cold, indulgence in ardent spirits, diuretic and irritating remedies, such as cantharides, violent exercise on horseback, venereal excesses; and it exists in connexion with hæmorrhoids, and other diseases of the rectum. In some of the injuries and diseases of the spine, this state of the bladder frequently occurs. Soemmering dwells strongly on suppressed gout as the cause of the complaint, a fancy evidently too far-fetched. The worst case which Dr. Prout ever saw was in a gouty subject.

"There are some habits apparently more predisposed to this affection than others; such are those of an irritable scrofulous temperament, with fair skin, and tendency to cutaneous affections, more especially if they have been accustomed to live freely, or been given to venereal excesses, or have suffered from venereal affections, or gout. In such individuals exposure to cold seems one of the most frequent causes of this affection, and those who actually labour under it are generally found to suffer much more severely in cold weather.

Cases of a milder character have been observed to terminate in a short time, or to assume an intermittent form, especially when associated with hæmorrhoids, or certain petechial affections. Old persons mostly retain the complaint as long as they live." 79.

*Treatment.*—If stone in the bladder is the cause of the complaint, this will not cease till the stone is removed, supposing that proper or practicable. In severe cases, there is generally retention of urine, and the water must be drawn off twice every twenty-four hours. Mr. Coulson quotes, and we quote again, the sentiments and directions of Sir Benjamin Brodie, with respect to injecting the bladder. Sir Benjamin observes that

"In aggravated cases of the disease, where the symptoms are at their greatest height, the mildest injections, even those of tepid-water, will do harm rather than good. They are especially to be avoided when the mucus deposited by the urine is highly tinged with blood. When, however, the symptoms have in some degree abated, the injection of tepid-water, or decoction of poppies, is in many instances productive of excellent effects. An elastic gum catheter may be introduced into the bladder, and the injection may be made by means of a small elastic gum syringe. The liquid should be allowed to remain in the bladder about thirty or forty seconds, and not more than an ounce and a half or two ounces should be injected each time. If the bladder be distended, so as to occasion any considerable degree of pain, the effect is always injurious instead of being beneficial. This operation may be repeated according to circumstances, once or twice in twenty-four hours. When there is a further abatement of the symptoms, the disease having assumed a still more chronic form, and the mucus being free, except on extraordinary occasions, from all admixture of blood, we may venture to add to the injection a very small quantity of nitric acid. At first, the proportion ought not to be more than that of one minim of the concentrated, or ten minims of the diluted nitric acid, to two ounces of distilled water; but afterwards this proportion may be doubled." 80.

The injection of the solution of nitric acid is also beneficial when the mucus deposits phosphate of lime, which combines with triple phosphate, furnished by the urine.

Mr. Coulson recommends as medicines, when there is much secretion of mucus, the decoction of the uva ursi with muriated tincture of iron, and small doses of powdered galls and nitre. The decoction of the Pareira Brava, combined with the nitric acid, is very useful also. The Pareira Brava, as our readers know, has re-appeared in our new Pharmacopœia, after a temporary absence from its pages. It was much in vogue in the beginning of the last century, and was lauded in a work published by Andreas Helvetius, as a specific for the affections of the bladder and kidney. Mr. Coulson has tried some experiments on the methods of preparing the remedy for use, which deserve attention.

"The mode of preparing this medicine, as advised in the present Pharmacopœia, is to put six drachms of the root into a pint of water and to macerate it for two hours. I usually, however, order a decoction—an ounce to a pint and a half of water, to be boiled to a pint.

Messrs. W. Allen and Co., of Plough-court, have recently made some experiments for me as to the advantage of macerating the roots previous to the boiling.

Three decoctions of the radix pareira brava were prepared with the following differences:—

1. Without previous maceration in cold water.
2. With previous maceration of 4 hours.
3. Ditto 12 hours.

On comparison, there appear but slight variations in the results. No. 3, however, seems to possess *rather a stronger taste* than No, 1. Perhaps the same may be said (in a less degree) of No. 2.

These decoctions are filtered with difficulty; but, by long standing, the feculent matter was separated, and the supernatant portions compared together. No. 1, was perfectly bright, while Nos. 2 and 3, were not quite so; yet, contrary to expectation, No. 1, was found to be of a rather greater specific gravity. This would imply that the previous cold maceration *does extract something, not permanently to remain in solution, but to be precipitated, and with it to carry down some other matter during the subsequent boiling.* If this be the case, it indicates the impropriety of the feculent part being separated—at least, until it is ascertained to possess no medicinal efficacy." 110.

If there is much pain or restlessness, morphium or opium should be exhibited—colchicum should be administered if there is any tendency to gout*—small doses of copaiba or the essential oil of cubebs with hyoscyamus, and with or without the pareira brava, may be useful, or may be just the contrary—light nourishment—warmth—the greatest care in all respects—such is the category of remedial measures.

### V. Acute Inflammation of the Muscular Structure of the Bladder.

Such is the subject and the title of the fifth chapter of Mr. Coulson's work.

Some authors and many surgeons doubt whether the muscular coat of the bladder is inflamed without the mucous and peritoneal tunics participating in the morbid action. Mr. Coulson, however, agrees with those who have considered that either of the tunics of the organ may be singly and separately affected. But he admits that acute idiopathic inflammation of the muscular tunic is comparatively rare, whilst affections of the mucous membrane, and chronic affections of the muscular tissue are frequent.

It more frequently, he says, attacks adults than the young or old, strong robust persons than the delicate, and males than females. The symptoms are given by Mr. Coulson.

The patient, he says, first complains of a dull aching pain in the region of the bladder, which soon becomes more violent and extends itself to the neighbouring organs. This pain is increased by pressure, and is attended by a desire to pass the urine, without, however, the power to satisfy it. The desire is not incessant, but comes on in paroxysms, attended with pain. The urine is evacuated at first in small quantities, and the attempts to pass it cause great pain. The small quantity which escapes is of a dark colour, sometimes not unlike coffee in appearance, at other times of a deep red, and even bloody colour, and at last complete retention occurs.

There is a sense of fulness in the lower part of the abdomen, pains in the lumbar region, in the groins, and down the thighs, but there is not the burning sensation along the urethra and in the perineum, which is found in the inflammation of the mucous membrane. The complaint is ushered in by rigors, which are soon succeeded by great constitutional disturbance. The pulse is full and hard, the thirst is great, and the skin hot, with general uneasiness and sickness. If the inflammation increases, pains are felt in the intestines, particularly in the rectum, combined with tenesmus, delirium comes on, the pulse rapidly sinks, and the patient soon dies. If the inflammation be seated, as is frequently the case, in the neck of the bladder, the urine, which has entered the bladder, is retained by the inflammatory action in that part, and the bladder soon becomes distended and projects above the pubes. There is a sense of weight in the perineum, and often painful erections of the penis, and an examination by the rectum gives great pain. These symptoms are explained by the anatomical structure and extreme sensibility of the trigone.

---

* A grain or two of the acetous extract may be given at bed time. We have tried this a good deal, as it has been much patronised by several eminent men, but we must own that we are not so satisfied of its virtues as we could desire.

If the inflammation is situated more in the upper part of the bladder, the desire to pass water is not so frequent, and the difficulty is less, but there is danger of implication of the peritoneum.

The progress of the complaint depends on its severity. Occurring after suppressed gout it may quickly terminate fatally, but, usually, after two or three days, if active measures are employed, the symptoms begin to yield.

"This form of inflammation sometimes terminates in the formation of abscesses in the coats of the bladder, and the symptoms are of a very formidable character, depending upon the size and situation of the abscess. The urgent symptoms of the inflammation subside; but there is a dull pain in the region of the bladder, occasional rigors, with febrile excitement, and uneasiness in passing the urine and the fæces. The abscess may open into the cavity of the bladder, in which case the pus is evacuated with the urine, and the patient experiences great relief; or, on the other hand, the matter may extend into the cellular tissue of the pelvis, and make its way either through the rectum, or to the perineum, or even to the groins, in which case the result is more frequently fatal. Mr. Wilson mentions an interesting case, where extensive suppurations had taken place in the coats from the prostate to the fundus of the bladder, the matter being lodged every where between the coats, and near the fundus several ulcerations had taken place through the internal membrane, by which the matter had passed into the cavity of the bladder." 98.

The complaint may be mistaken for acute inflammation of the prostate gland, but, in the latter, there is more fulness and tenderness on pressure in the perineum, and, on examination, per rectum, the prostate is found exceedingly painful and sensitive.

The morbid appearances are, as might be expected, great injection of the muscular coat with blood—a dark colour, sometimes even a gangrenous condition of it. The mucous membrane is also of a dark red colour. In other cases, the membrane will be found thickened, and the bladder itself contracted. Pus is found sometimes infiltrated through the tunic, or else circumscribed in the form of an abscess.

The causes of the complaint are exposure to cold—indulgence in spirituous liquors—more frequently gonorrhœa—wounds, the incautious use of instruments, or other local injuries—the immoderate use or even the external application of cantharides—irregular or suppressed gout.

Such is Mr. Coulson's account of acute inflammation of the muscular coat of the bladder. We must say that we have never seen a case in which we were satisfied of the limitation of the inflammation to this coat. That inflammation may in one instance be principally seated in one tunic, and that in another instance it may be mainly located in another tunic, is too certain to admit of doubt; but its absolute limitation to one coat, and particularly to a central coat, like the muscular, does not seem to us so well established in fact, as in theory. The only case related by Mr. Coulson as a sample of acute inflammation of the muscular coat of the bladder, displayed, on dissection, the signs of inflammation in all the coats. Mr. Coulson examined the body on the day after the decease, and "found the bladder in an intense state of inflammation: there was no organic change in its structure, but the tunics, particularly the muscular coat, were of a very deep and red colour." But whilst we are sceptical, at present, of the separate existence of acute inflammation of the muscular coat of the bladder, we are sensible of the utility of directing attention to the possibility of its occurrence, and of awakening attention to the varieties of symptoms which a greater or less affection of any particular tunic of the organ may give rise to.

*Treatment.*—Prompt measures are requisite in a disorder so acute as this. General blood-letting, if the patient be strong or robust, must be first employed; or, if the patient be delicate, and of a spare habit, local bleeding, as leeches to the pubes, or cupping in the perineum, may be substituted; or these may be regarded as auxiliaries to the general blood-letting.

Hot fomentations should be constantly applied to the pubes, and the patient, after the bleeding, should be placed in a hot bath. As there is retention of urine, the latter must be drawn off from time to time by the catheter. Calomel and opium—sedative injections or suppositories—saline aperients with colchicum, especially if the attack is of a gouty character—and warm mucilaginous drinks, are the remedies which seem most adapted for the case.

### VI. Chronic Inflammation of the Muscular Coat of the Bladder.

The sixth chapter is occupied with this affection. It may supervene on the acute form, or may occur independently of it altogether.

In this complaint, says Mr. Coulson, there is uneasiness about the region of the bladder, frequent desire to make water, both by night and day, but especially by night, and the urine does not flow so readily as it did; there is also, occasionally, prolapsus of the rectum. The patient frequently complains of pains in all the limbs and in the region of the back; the skin is dry and attacked with psoriasis or lepra; the urine is scanty, of deep colour, and of high specific gravity. The kidneys, after a time, are involved in the progress of the disease, the urine becomes albuminous, nausea supervenes, the patient loses flesh and strength, and sinks at last from complete exhaustion.

The coats of the bladder becoming thick and hard, and no longer admitting of the former degree of extension, the patient feels a sense of weight in the pelvis, and the bladder may be felt through the rectum as a hard thick body. The patient is unable to empty the organ completely, and the small remaining quantity is a source of great irritation, and of extreme efforts to expel it.

On dissection, the bladder will be found more or less thickened; and its inner surface presents a considerable number of rugæ, caused by the projection of the enlarged fasciculi beneath. The thickness of the bladder is sometimes very considerable. Dr. Baillie has given the representation of a bladder nearly an inch in thickness: the prostate gland is enlarged.

"This disease is frequently the sequel of acute inflammation, but is also caused by strictures in the urethra, enlargement of the prostate, prostatic calculi, cold, stone, indulgence in spirituous liquors, irritating medicines, as cantharides; and in some constitutions the long-continued use of cubebs and copaiba. In persons, in whom there is an hereditary predisposition to urinary affections, it occurs from slight causes, as well as in gouty and rheumatic subjects. The retention of the urine in the bladder, after the desire to void it has been felt, often brings on this complaint.

This affection is likely to be confounded with simple irritation of the bladder, but the absence of pain, and of the constitutional symptoms which I have described, is the great diagnostic sign. Hysterical females are also subject to a peculiar form of irritation of the bladder, which might be mistaken for inflammation. They suffer great pain, and even have retention of urine; but the temperament or constitution of the patient will shew the nature of the disease." 108.

Mr. Coulson remarks that any obstacle to the issue of urine from the bladder will give rise to hypertrophy of the muscular fibres—a hypertrophy which must not be deemed the consequence of inflammation.

*Treatment.* The first thing is to determine the cause, and to remove it, supposing its removal possible. The complaint itself is extremely troublesome. Mr. Coulson recommends, in gouty, rheumatic, or plethoric persons, colchicum given at night, in the dose of one or two grains of the acetous extract. As the urine is acid and often scanty, Mr. C. advises the alkalies to be given after meals, in a form often used by Dr. Prout, viz. the bicarbonate of potass, sesquicarbonate of soda, and nitrate of potass. He recommends, too, the pareira brava.

"The diosma, in the form of an infusion, combined with the alkalies and tincture of hyoscyamus, will be found of great service. Should the urine not be acid, or, as is not unfrequently the case, should the alkalies produce headache and restlessness, or uneasiness about the region of the stomach, then their use must be discontinued, and recourse had only to sedatives, as the extract of hop, and extract of uva ursi, or the nitric ether, with the tinctura camp. co.; and the occasional exhibitions of suppositories. The diet should be plain, but nutritious, and all beer, wine and spirits should be prohibited. Exposure to wet and cold invariably aggravate this complaint, and should, of course, be avoided." 113.

### VII. Inflammation of the Peritoneal Coat of the Bladder, and of the surrounding Cellular Tissue.

The peritoneal coat of the bladder may be inflamed without the other coats being involved, in consequence of the laxity of the cellular connexion between it and the muscular tunic. Dr. Todd observes, that there are on record two cases in which acute inflammation was limited to the peritoneal tunic of the organ. The inflammation of the peritoneal coat of the bladder seldom fails to extend to other parts of the general peritoneum. It is often the close of a fatal disease of the bladder, but the old adhesions often found between the bladder and other viscera shew that the affection is often not so serious.

The pain and its aggravation on pressure, the state of the pulse, the countenance, and position of the body, clearly indicate the nature of the complaint.

The treatment is that adapted for peritonitis, modified by the particular circumstances of the case, whatever they may happen to be.

Mr. Coulson just touches on a severe but highly interesting affection—diffuse inflammation of the cellular membrane of the pelvis. But he only touches on it, and we should not, therefore, think ourselves justified in going into it.

VIII. Fungus Hæmatodes and Cancer of the Bladder.

Mr. Coulson does not add much to what was already known with respect to these affections. We shall therefore say little about them.

Fungous excrescences, he observes, occasionally arise from the inner surface of the bladder, and are productive of most distressing symptoms, often very similar to many of those which attend the stone. These excrescences are different in their size, and in the extent of surface which they occupy. Sometimes they originate from a single root, and occasionally from several; but they generally consist of a similar loose and fibrous structure. In certain situations, as when immediately behind the neck of the bladder, they will, by blocking up the origin of the urethra, cause a very considerable obstruction to the passage of the urine; and the bladder being irritated, and frequently excited by them to stronger action than in a natural state, its muscular coat becomes thickened. These excrescences are sometimes attended with a discharge of blood, and of a viscid ropy mucus, the result of irritation of the inner membrane of the bladder, and with pain along the urethra, and at the glans penis. The glands in the groins and pelvis, usually, become enlarged.

The fungus is often of the true medullary kind, breaking and bleeding with facility. There is always a disposition to hæmorrhage, and the urine frequently contains small portions of the medullary matter, the latter being the most distinctive sign of the disease.

The treatment is so exclusively palliative, that we do not consider it requisite to dwell on it. Mr. Coulson very judiciously insists on the necessity for the horizontal posture. We extract one remark on albuminous urine, which merits the attention of our readers.

"The powers of life are generally much exhausted; and the urine is often alkaline, containing albumen, and a large proportion of the phosphates.

I may, perhaps, observe here, that when albuminous urine is alkaline, it is sometimes incapable of being coagulated by heat. It was supposed that this depended on the presence of some fixed alkali, which held the albumen in solution. Mr. Rees made an analysis of two different specimens of urine taken from the same individual: one was neutral, and coagulable by heat; the other was not coagulable by heat, and possessed an alkaline re-action. From the analysis it appeared, that the alkaline specimen contained the greatest proportion of albumen, and a much smaller proportion of alkaline salts than the neutral urine. This goes strongly, he observes, against the probability of any fixed alkali, being the solvent of the albumen; for in this case we should expect a redundant quantity of fixed saline matter, in proportion to the albumen present, whereas, exactly the opposite, was the result." 132.

IX. Foreign Bodies in the Bladder; Operation for Stone.

The chapter is a brief one, and a few observations are all that we shall extract from it.

Mr. Coulson alludes to several recorded instances of hair voided with the urine. The most curious, perhaps, is one quoted by Mr. Howship, and which occurred to Dr. Wallace. In this case, hair was several times voided with the urine; and on the body, after death, being examined, a stone was found in the bladder, large as a goose egg, from parts of which hairs had grown out. It was thought that the hairs voided during life, which were a great many, and some of an extraordinary length, grew out of that stone; because when the hairs hung out of the urethra, as they frequently did, to his great torment, they were obliged to be pulled out, which was always done, with a resistance, as if plucked out by the root. It is evident that some fallacy exists in this observation, though it would be useless now speculating on what that fallacy was.

Musket-balls have found their way into the bladder. So have female catheters. The following case quoted from Ingleby may offer a hint on the mode of removing it.

"The patient was in the fourth month of pregnancy, and had experienced a retention of urine, by no means uncommon, just before the uterus finally quits the pelvis for the abdomen, but in this instance occasioned by the womb being considerably prolapsed—a circumstance which it is material to mention. At the time I saw the patient, the catheter had been in the bladder eight hours. It lay in the centre of the organ, quite transversely; and, the urine having dribbled away, the bladder was in as contracted a state as the catheter admitted of. By means of a long and very slender pair of forceps passed per urethram, I embraced the instrument near one end, and with the two fore-fingers of the left hand passed by the vagina, carefully elevated the other end; and having thus brought it into the horizontal direction, gently extracted it." 136.

On the third day, the ovum was discharged without hæmorrhage, and under the skin of the scalp of the fœtus was a considerable extravasation of blood.

Mr. Toogood, of Bridgewater, has recently published in the Medical Gazette, two interesting cases, where the female catheter slipped into the urethra. In both cases, the instruments were extracted by dilating the urethra by the sponge-tent, so as to enable the fore-finger to be introduced into the bladder.

"Mr. Key and myself saw, two or three years ago, a case, where three inches of a thin gum elastic catheter broke in the urethra, near to the bladder, and although we were called to the gentleman immediately after the occurrence of the accident, we could not lay hold of the broken portion. Three weeks after this, the fragments (for there were two, one an inch in length, and the other two inches) were voided by the urethra. From the fortunate termination of this case, I came to the determination always to wait, in similar cases, before recommending an operation." 137.

Mr. Tyrrell has lately published a case, in which a catheter broke in the urethra and escaped into the bladder. He extracted it by Weiss's forceps. We have detailed the particulars of the case in a former number of this Journal.

Hydatids and worms have been discharged from the bladder. We need not do more than mention the fact. We are unable to notice some interesting remarks on the method of performing the operation of lithotomy, with which Mr. Coulson concludes the chapter.

The tenth and last chapter, on Wounds and Injuries of the Bladder, does not seem to require particular notice. We therefore take our leave of this work and of its author—the former fraught with much excellent practical information—the latter a zealous, intelligent, and skilful surgeon, and a gentleman possessed of the most honourable feelings. We wish both the success which they merit, and which we are confident they will obtain.

